# Effect of Weed Management and Seed Rate on Crop Growth under Direct Dry Seeded Rice Systems in Bangladesh

**DOI:** 10.1371/journal.pone.0101919

**Published:** 2014-07-07

**Authors:** Sharif Ahmed, Muhammad Salim, Bhagirath S. Chauhan

**Affiliations:** 1 Crop and Environmental Sciences Division, International Rice Research Institute, Metro Manila, Los Baños, Philippines; 2 Agronomy Division, Bangladesh Agricultural University, Mymensingh, Bangladesh; 3 Queensland Alliance for Agriculture and Food Innovation (QAAFI), The University of Queensland, Toowoomba, Queensland, Australia; California State University, Fresno, CA, United States of America

## Abstract

Weeds are a major constraint to the success of dry-seeded rice (DSR). The main means of managing these in a DSR system is through chemical weed control using herbicides. However, the use of herbicides alone may not be sustainable in the long term. Approaches that aim for high crop competitiveness therefore need to be exploited. One such approach is the use of high rice seeding rates. Experiments were conducted in the aman (wet) seasons of 2012 and 2013 in Bangladesh to evaluate the effect of weed infestation level (partially-weedy and weed-free) and rice seeding rate (20, 40, 60, 80, and 100 kg ha^−1^) on weed and crop growth in DSR. Under weed-free conditions, higher crop yields (5.1 and 5.2 t ha^−1^ in the 2012 and 2013 seasons, respectively) were obtained at the seeding rate of 40 kg ha^−1^ and thereafter, yield decreased slightly beyond 40 kg seed ha^−1^. Under partially-weedy conditions, yield increased by 30 to 33% (2.0–2.2 and 2.9–3.2 t ha^−1^ in the 2012 and 2013 seasons, respectively) with increase in seeding rate from 20 to 100 kg ha^−1^. In the partially-weedy plots, weed biomass decreased by 41–60% and 54–56% at 35 days after sowing and at crop anthesis, respectively, when seeding rate increased from 20 to 100 kg ha^−1^. Results from our study suggest that increasing seeding rates in DSR can suppress weed growth and reduce grain yield losses from weed competition.

## Introduction

As in many Asian countries, conventional puddled-transplanted rice (CPTR) is the major system of rice production in Bangladesh. This system is becoming less profitable and less sustainable because of its high labor, water, and energy requirements. Agricultural laborers are becoming involved in other non-farm jobs such as textile, garments, and other industries. As a result, it is often difficult to find labor during transplanting. In addition, the level of groundwater, the major source of irrigation for cultivation has been declining due to excessive withdrawal. [1]. It has been predicted that a significant amount of rice area in Asia may suffer from physical and economic water scarcity by 2025 [2]. To overcome water and labor problems, farmers in many Asian countries including Bangladesh are increasingly adopting dry-seeded rice (DSR) production systems.

DSR has several advantages over CPTR. DSR systems save labor by eliminating the need for managing a nursery bed, seedling uprooting, puddling, and transplanting. In DSR fields, there is no need to keep standing water at all the growth stages, which helps save a huge amount of water compared with CPTR fields [3]. DSR systems can also be harvested at least 7–10 days before CPTR systems [4], which facilitates the timely planting of subsequent crops [e.g., wheat (*Triticum aestivum* L.), mustard (*Brassica juncea* (L.) Czern), lentil (*Lens culinaris* Medic.), and potato (*Solanum tuberosum* L.)]. In DSR, however, weeds are a major constraint to achieving high yield [5]. The main reasons for the heightened weed problem in DSR are that weeds and rice seedlings emerge simultaneously, which reduces the competitive advantage of the crop, and that alternate events of wetting and drying enhances the growth of weeds [6]. If weeds are not controlled in DSR, yield losses could exceed 90%, depending on factors such as tillage system, type of cultivar, seeding rate, water management, types of weed flora, and field conditions [7]. Timely weed management is thus crucial to the success of DSR.

In Bangladesh, weeding is commonly done manually. The practice, however, is becoming less common due to labor scarcity. In recent years, chemical weed control has increased in Bangladesh, but reliance only on herbicides is not sustainable as the continuous use of the same mode of action over a long period may result in the evolution of herbicide-resistant weed biotypes [8], [9], [10]. In addition, it is not possible to control a range of weeds using a single herbicide. Weed management in DSR therefore needs an integrated approach [7], [11], [12].

Higher seeding rate is one approach that helps increase crop competitiveness against weeds [7], [13], [14]. High seeding rates facilitate quick canopy closure, which helps suppress weeds more effectively. At low seeding rates, crop plants take more time to close their canopy, which encourages weed growth [15]. High seeding rates improve the ability of crops to suppress weeds and can reduce yield loss under partially-weedy conditions. Similar results have been reported elsewhere for different crops, including barley (*Hordeum vulgare* L.) [16], wheat [17], and soybean [*Glycine max* (L.) Merr] [18]. In the Philippines, seeding rates of 100–300 viable seeds m^−2^ increased yield and decreased weed biomass in aerobic rice systems [19]. In lowland rice, higher seeding rates favored rice against weeds and, at the same time, increased yields under weedy conditions [20].

Studies in different countries suggest that crop seeding rate can affect weed growth and rice grain yield. Such information, however, is not available in Bangladesh. The objective of this study was, therefore, to evaluate the effect of weed management and seeding rate on weed density, weed biomass, crop yield, and yield components in DSR.

## Materials and Methods

The authors confirm that no permission was needed to conduct the experiment, as well as that the field studies did not involve endangered or protected species.

**Figure 1:**
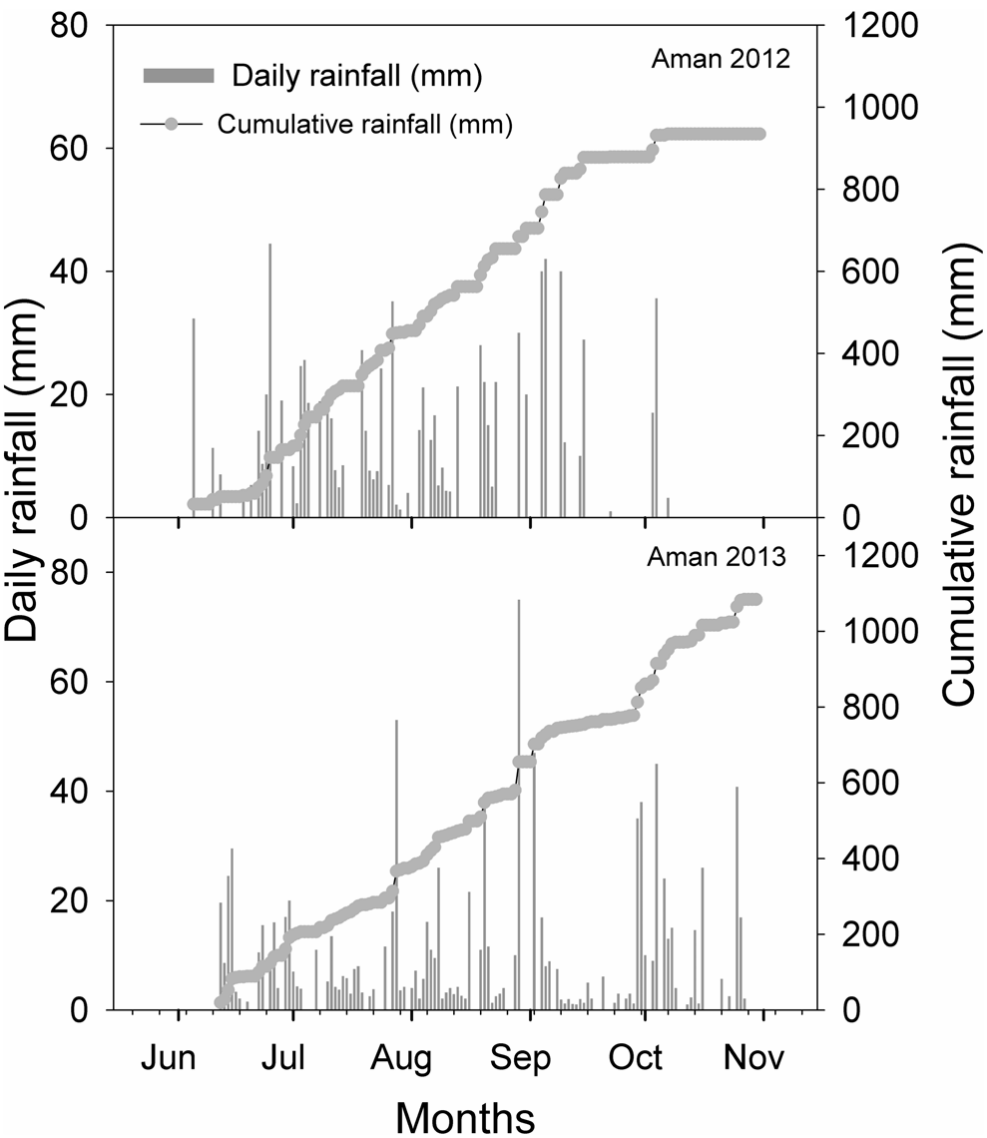
Daily and cumulative rainfall during the aman seasons of 2012 and 2013.

Field experiments were conducted during the aman seasons (June to October) of 2012 and 2013 at the Regional Agricultural Research Station (RARS) of the Bangladesh Agricultural Research Institute (BARI) in Jessore (23°11′ N, 89°14′ E; 16 m above mean sea level). Historically, the area is known as the High Ganges River Floodplain and is predominantly highland. The climate of the area is subtropical, with an average annual rainfall of 1,590 mm, minimum temperatures of 6–9°C in January, and maximum temperatures of 36–44°C in April and May. Rainfall recorded at the site during the experimental periods is presented in Figure 1. Soil in the experimental fields at 0–15-cm depth was clay loam in texture with a bulk density of 1.60 Mg m^−3^, pH of 7.8, organic carbon of 1%, sand of 31%, silt of 31%, and clay of 38%. The experimental area was dry-cultivated using a four-wheel tractor before crop sowing. The experiments in each year were arranged in a split-plot design with weed infestation level (partially-weedy and weed-free) in the main plots and seeding rate (20, 40, 60, 80, and 100 kg ha^−1^) in the subplots. The area of each subplot was 13.5 m^2^; three replications were made in each season. The rice cultivar BRRI dhan49 (135-d duration) was used in both seasons.

Dry rice seeds were sown by hand at different seeding rates with row spacing of 20 cm. In both years, the crop was sown on June 20 and harvested at the end of October. Fertilizers were applied at the rates of 120-15-48-12-2.2 kg ha^−1^ of N, P, K, S, and Zn in the form of urea, triple superphosphate (TSP), muriate of potash (MoP), gypsum, and zinc sulphate, respectively. Full doses of TSP, MoP, gypsum, and zinc sulphate were applied immediately before sowing. Urea was applied in four equal splits at 14 d after sowing (DAS), at the start of tillering (35 DAS), at maximum tillering (50 DAS), and at panicle initiation (70 DAS).

In weed-free plots, weeds were managed initially by the application of 80 g ai ha^−1^ of oxadiargyl (Topstar 400 SC, Bayer Crop Science Limited, Bangladesh) at 2 DAS and later with hand weeding (at 15, 25, 35, 50 and 70 DAS). The herbicide was applied using a knapsack-sprayer, attached with three flat-fan nozzles on a boom that delivered 450 L solution ha^−1^. In the partially-weedy plots, emerged weeds were manually removed once at 36 DAS and then weeds were allowed to grow for the rest of the season. One hand weeding was done in these plots because allowing weeds to grow throughout the season could have resulted in more than 90% yield loss in DSR systems [7]. In addition, it is not common for farmers in irrigated areas to leave their rice fields infested with weeds throughout the season.

The field was irrigated lightly immediately after sowing, after which irrigation was made based on tensiometer readings using a threshold value of 15 kPa at 15 cm soil depth. At each irrigation, water was added until its depth on the soil surface reached 5 cm. At 50 DAS, 300 g ai ha^−1^ of the fungicide tebuconazole + trifloxystrobin 75 WP (Nativo 75 WP, Bayer Crop Science Limited, Bangladesh) was applied and, at 70 DAS, 300 g ai ha^−1^ of fipronil 3G (Regent 3GR, BASF Bangladesh Limited) was applied to control blast and stem borers, respectively.

Rice plant density was evaluated at 14 DAS from four randomly selected 1-m row lengths in each plot. Weed density and weed biomass were measured at 35 DAS and at anthesis. At each sampling time, two quadrats of 40 cm×40 cm were placed randomly in each plot and weeds were collected from each quadrat. Collected weeds were clustered by group (i.e., grass, broadleaf, and sedges) and counted. Weed biomass was measured group-wise after the samples were oven-dried at 70°C for 72 h. Weed density and biomass data were converted to density or biomass per m^2^. The number of rice tillers and rice dry biomass were determined through collection of samples from the same quadrats used for weed sampling, and on the same date. At harvest, rice panicles were counted from four randomly placed 1-m row lengths in each plot. Grains per panicle (filled and unfilled) were counted by randomly sampling 20 panicles per plot. Rice grain yield was determined from the harvested area of 8.8 (4.0 m×2.2 m) m^2^. Grain yield was converted to t ha^−1^ at 14% moisture content. Data were analyzed using ANOVA to evaluate differences between treatments and the means were separated using least significant differences (LSD) at 5% level of significance (Crop Stat 7.2; International Rice Research Institute, Philippines). In a combined analysis of data, the interactions of years with the treatments were significant therefore, the data were presented separately. Weed density and biomass data were transformed using square-root transformation (√x+0.5) before analyses. Transformation, however, did not improve homogeneity. Therefore, original values were used for analysis and presentation. The relationship of seeding rate (kg ha^−1^) with weed biomass (g m^−2^) and rice biomass (g m^−2^) were assessed using linear regression analysis (SigmaPlot 11, Systat Software Inc.). In the regression analysis, the values of all individual replications were included.

## Results and Discussion

### Rice plant density

**Figure 2:**
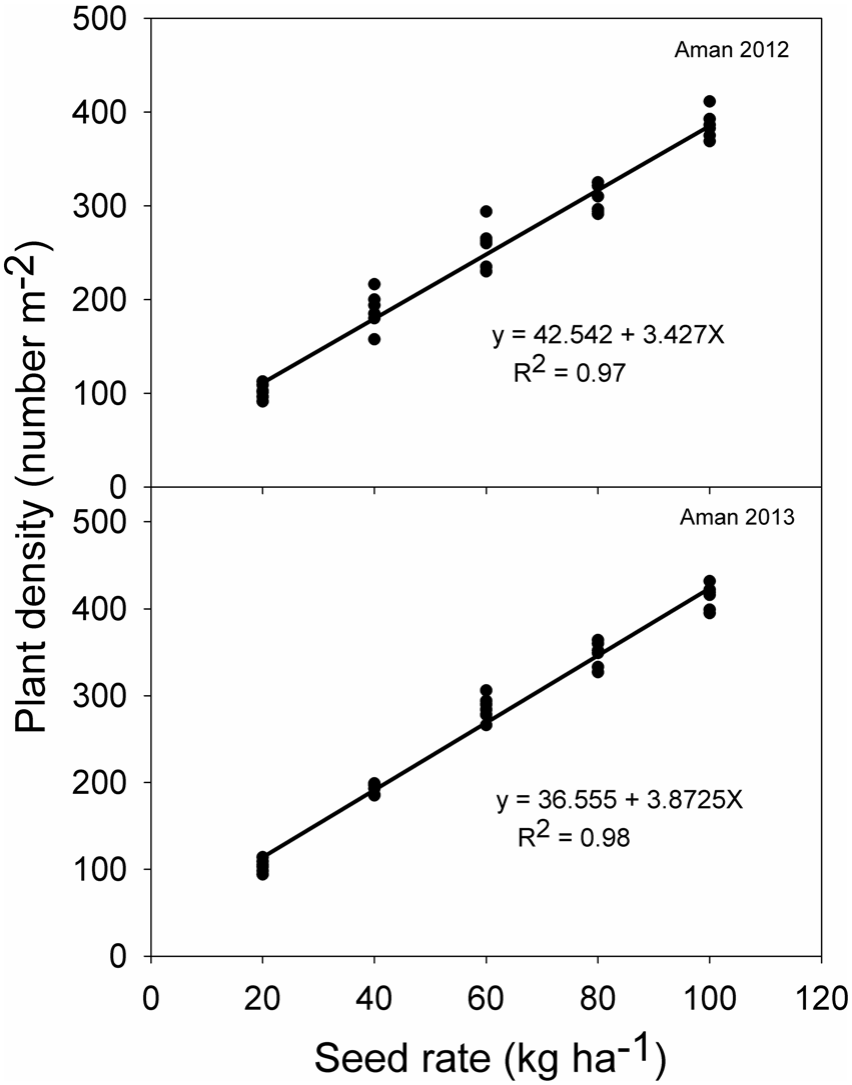
Relationship between rice seeding rate and rice plant density at 14 days after sowing in 2012 and 2013.

Rice plant density was not influenced by weed infestation level but it was strongly influenced by seeding rate in both seasons (Table 1). Rice plant density ranged from 100 to 397 plants m^−2^ in 2012 and 100 to 415 plants m^−2^ in 2013 (data not shown). Plant density increased linearly with increase in seeding rates in both years (Figure 2).

**Table 1:** Analysis-of-variance results for year, weed infestation level (weed) and seed rate and their interactions on plant density, rice biomass at 35 DAS and at anthesis, rice panicle m^−2^, grains panicle^−1^, and grain yield.

Source of variation	Degree of freedom	*P*-value					
		Plant density (no. m^−2^)	Rice biomass at 35 DAS	Rice biomass at anthesis	Panicle m^−2^	Grains panicle^−1^	Grain yield (t ha^−1^)
Year	4	0.01	0.01	0.02	0.01	0.25	0.04
Weed	1	<0.001	<0.001	<0.001	<0.001	<0.001	<0.001
Year×weed	1	0.05	0.12	0.01	0.19	0.09	0.02
Seed rate	4	<0.001	<0.001	<0.001	<0.001	0.30	<0.001
Weed×seed rate	4	0.82	<0.001	<0.001	0.59	<0.001	0.001
Year×seed rate	4	<0.001	0.02	0.86	0.53	0.95	0.74
Year×weed×seed rate	4	0.70	0.03	0.94	0.91	0.91	0.09

### Weed density and biomass

The common weed species found at the experimental site were tropic ageratum (*Ageratum conyzoides* L.), spiny amaranth (*Amaranthus spinosus* L.), scarlet pimpernel (*Anagalis arvensis* L.), fringed spider-flower (*Cleome rutidosperma* D.C.), celosia (*Celosia argentea* L), bermudagrass [*Cynodon dactylon* (L.) Pers.], purple nutsedge (*Cyperus rotundus* L.), crowfootgrass [*Dactyloctenium aegyptium* (L.) Willd.], southern crabgrass [*Digitaria ciliaris* (Retz.) Koel.], junglerice [*Echinochloa colona* (L.) Link], goosegrass [*Eleusine indica* (L) Gaertn], fringed quickweed [*Galinsoga ciliata* (Raf.) Blake], and niruri (*Phyllanthus niruri* L.). Results are presented for each weed group (i.e., grass, broadleaf, and sedges).

At 35 DAS (before hand weeding in the weedy plots), total weed density was influenced (P<0.05) by seeding rate (Table 2). Higher seeding rate resulted in lower weed density compared to the lowest seeding (20 kg ha^−1^) (Table 3). With increase in seeding rate from 20 to 100 kg ha^−1^, weed density decreased by 38% and 47% in the 2012 and 2013 seasons, respectively. Similar to weed density results at 35 DAS, weed biomass was also influenced by seeding rate (Table 4). Weed biomass decreased significantly (by 54% and 56% in 2012 and 2013, respectively) when seeding rate increased from 20 to 100 kg ha^−1^. Grass weeds were found to be the dominant group in this study. The density and biomass of grass weeds decreased by 46–51% and 56–64% in 2012 and 2013, respectively, when seeding rate was increased to 100 kg ha^−1^ from 20 kg ha^−1^.

**Table 2:** Analysis-of-variance results for year and seed rate and their interactions on weed density and weed biomass at 35 DAS.

Source of variation	Degree of freedom	*P*-value							
		Density (no. m^−2^)				Biomass (g m^−2^)			
		Grass	Broadleaf	Sedges	Total	Grass	Broadleaf	Sedges	Total
Year	1	0.94	0.03	<0.001	<0.001	<0.001	0.86	0.01	0.02
Seed rate	4	4.02	0.37	0.11	<0.001	<0.001	<0.001	0.15	<0.001
Year×seed rate	4	0.06	<0.001	0.02	0.14	0.01	0.92	<0.001	0.56

**Table 3:** Effect of rice seeding rate on weed density (number m^−2^) at 35 days after sowing in the aman seasons of 2012 and 2013.

Seed rate(kg ha^−1^)	Weed density							
	Grass		Broadleaf		Sedges		Total	
	2012	2013	2012	2013	2012	2013	2012	2013
	number m^−2^							
20	631.3	575.0	47.9	98.9	45.8	328.1	725.0	1002.1
40	475.0	444.8	46.8	109.4	68.8	319.8	590.6	873.9
60	465.6	430.2	62.5	71.8	50.0	239.6	578.1	741.7
80	422.9	362.5	55.2	69.8	93.8	164.6	571.9	596.9
100	306.3	313.5	69.8	54.2	73.9	161.5	450.0	529.2
LSD_0.05_	111.2	NS	NS	32.3	40.8	144.3	106.9	245.2

Abbreviations: LSD_0.05_, least significant difference at 5% level of significance; NS, non-significant.

**Table 4:** Effect of rice seeding rate on weed biomass (g m^−2^) at 35 days after sowing in the aman seasons of 2012 and 2013.

Seed rate(kg ha^−1^)	Weed biomass							
	Grass		Broadleaf		Sedges		Total	
	2012	2013	2012	2013	2012	2013	2012	2013
	g m^−2^							
20	107.4	59.9	19.1	19.1	8.4	42.6	134.9	121.7
40	91.2	43.3	18.0	15.3	11.8	40.1	121.0	98.6
60	76.9	39.2	14.7	12.7	5.9	35.4	97.6	87.3
80	54.3	31.7	6.5	8.3	20.7	21.1	81.6	61.1
100	38.6	26.5	9.1	8.6	14.8	17.8	62.5	52.9
LSD_0.05_	15.1	19.7	10.1	6.5	7.53	17	11.9	18.3

Abbreviations: LSD_0.05_, least significant difference at 5% level of significance; NS, non-significant.

The effect of seeding rate on weed density and biomass at anthesis are given in Tables 5 and 6. Similar to observations made at 35 DAS, total weed density and biomass was greatly influenced by seeding rate at anthesis (Table 7). However, the effects of seeding rate on density and biomass of broadleaf and sedges were not significant at this stage. In 2012, total weed density and biomass decreased by 40 and 41%, respectively, when seeding rate increased from 20 to 100 kg ha^−1^; these values were 65 and 60%, respectively, in 2013.

**Table 5:** Effect of rice seeding rate on weed density (number m^−2^) at crop anthesis in the aman seasons of 2012 and 2013.

Seed rate(kg ha^−1^)	Weed density							
	Grass		Broadleaf		Sedges		Total	
	2012	2013	2012	2013	2012	2013	2012	2013
	number m^−2^							
20	233.3	256.3	82.3	78.1	3.1	20.8	318.8	355.2
40	221.9	182.3	52.1	75.0	2.1	10.4	276.0	267.7
60	158.3	196.9	43.8	47.9	0.0	0.0	202.1	244.8
80	178.1	131.3	39.6	42.7	0.0	0.0	217.7	173.9
100	146.9	104.2	41.7	15.6	0.0	3.1	188.5	122.9
LSD_0.05_	NS	100.7	NS	NS	NS	NS	90.8	127.2

Abbreviations: LSD_0.05_, least significant difference at 5% level of significance; NS, non-significant.

**Table 6:** Effect of rice seeding rate on weed biomass (g m^−2^) at crop anthesis in the aman seasons of 2012 and 2013.

Seed rate(kg ha^−1^)	Weed biomass							
	Grass		Broadleaf		Sedges		Total	
	2012	2013	2012	2013	2012	2013	2012	2013
	g m^−2^							
20	218.8	156.9	33.8	50.2	1.4	3.2	254.0	210.4
40	182.8	126.9	25.6	41.3	0.6	3.1	208.9	171.2
60	143.4	108.5	31.6	38.2	0.0	0.0	175.0	146.7
80	149.2	83.5	14.3	32.5	0.0	0.0	163.5	115.9
100	133.7	70.8	16.6	13.1	0.0	0.7	150.3	84.6
LSD_0.05_	58.3	41.2	NS	NS	NS	NS	57.1	34.8

Abbreviations: LSD_0.05_, least significant difference at 5% level of significance; NS, non-significant.

**Table 7:** Analysis-of-variance results for year and seed rate and their interactions on weed density and weed biomass at anthesis.

Source of variation	Degree of freedom	*P*-value							
		Density (no. m-2)				Biomass (g m^−2^)			
		Grass	Broadleaf	Sedges	Total	Grass	Broadleaf	Sedges	Total
Year	1	0.05	1.00	0.26	0.03	<0.001	0.04	0.81	0.01
Seed rate	4	0.01	0.14	0.39	<0.001	<0.001	0.11	<0.001	<0.001
Year×seed rate	4	0.42	0.81	0.69	0.82	0.86	0.80	0.44	0.77

**Figure 3:**
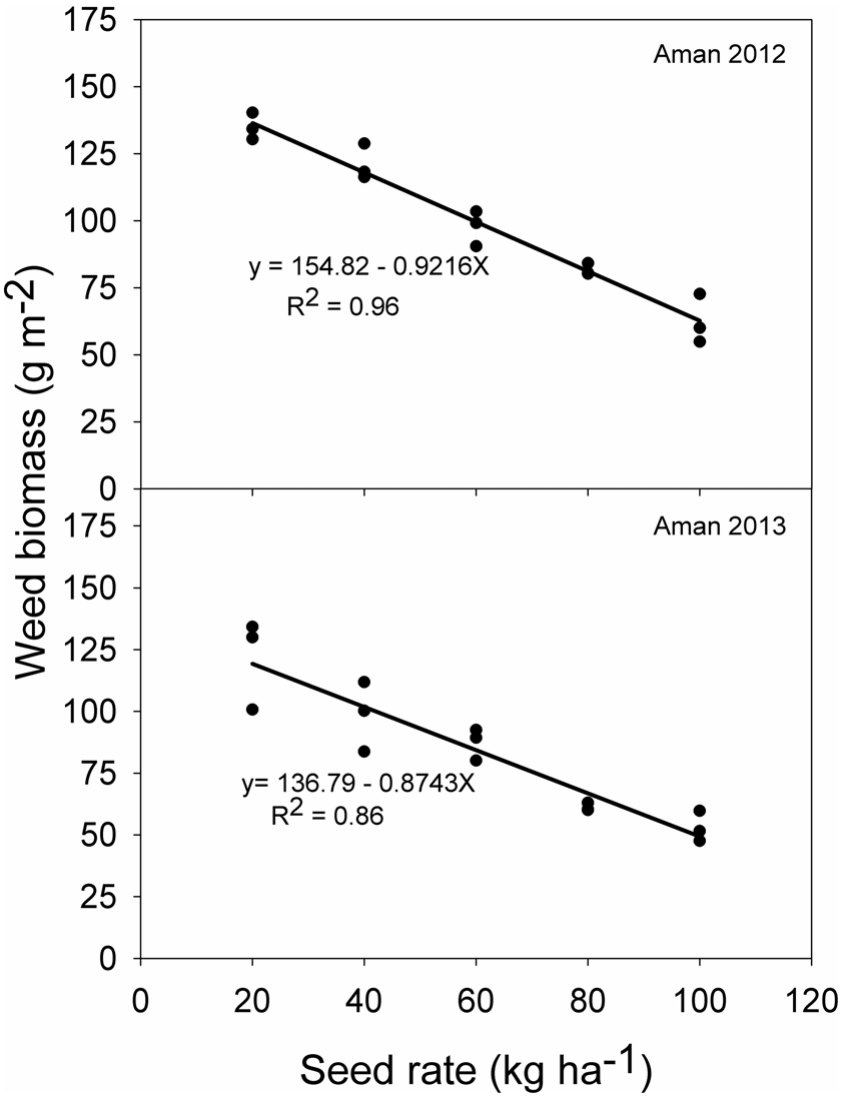
Relationship between rice seeding rate and weed biomass at 35 days after sowing in 2012 and 2013.

**Figure 4:**
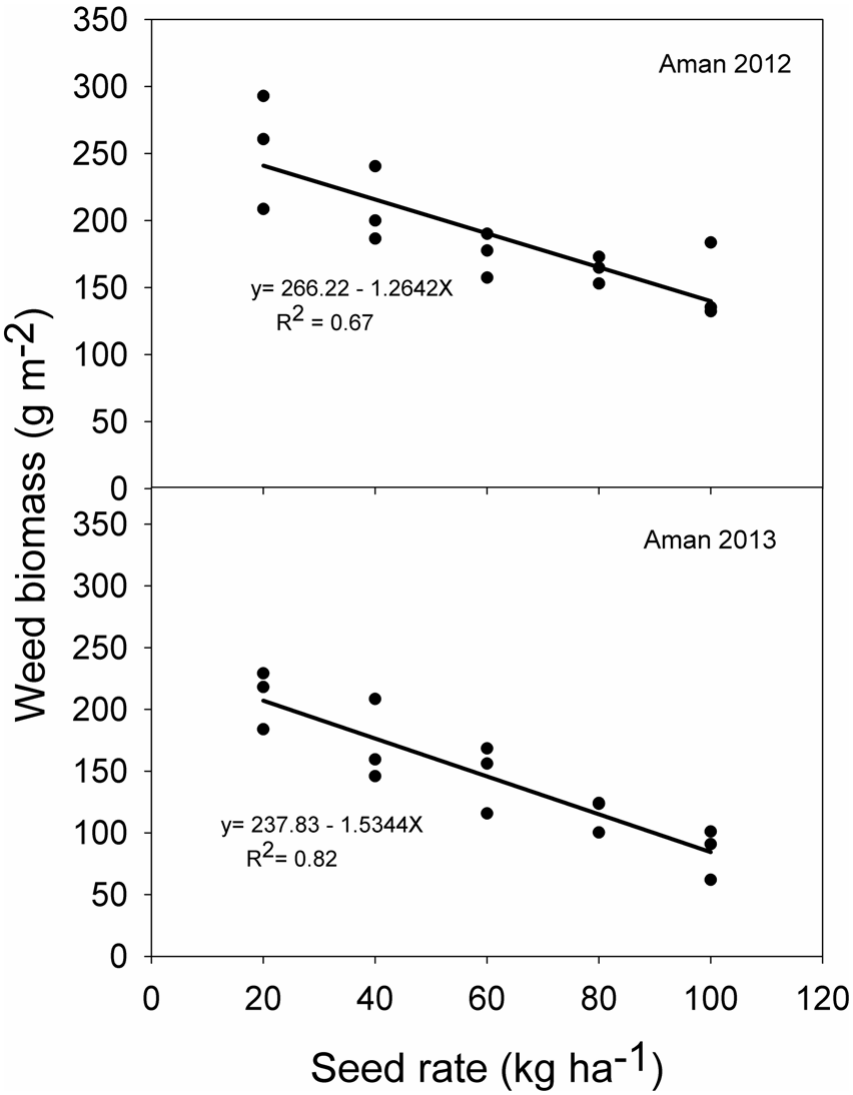
Relationship between rice seeding rate and weed biomass at crop anthesis in 2012 and 2013.

The relationship between rice seeding rate and weed biomass are shown in Figures 3 and 4. In both seasons, weed biomass decreased linearly with increasing seeding rate. This explained 96% and 86% of variations at 35 DAS in 2012 and 2013, respectively. At anthesis, these values were 67% and 82%, respectively.

At low seeding rates, weeds may have had a favorable environment that provided them an opportunity for easier germination and faster growth and development. But at high seeding rates, the crop had a competitive advantage over weeds and closed canopy earlier, thus, reducing weed growth. [20], [21], [22]. A previous study explained that, due to increased crop density; the crop fraction of the total plant biomass (crop + weed) was increased, which resulted in higher weed suppression [23]. It has been reported that fresh weight of grass weeds was reduced by 18% when seeding rate of wheat was doubled from the recommended rate (80 kg ha^−1^) [24]. Similarly, another study in an aerobic rice system found greater weed suppression when the seeding rate of rice was increased from 50 to 75 kg ha^−1^ [25].

### Rice biomass

Rice biomass was strongly influenced by weed infestation level and seeding rate (Table 8). At 35 DAS, rice biomass increased significantly when seed rate increased from 20 to 100 kg ha^−1^. At crop anthesis, however, rice biomass increased only up to the seeding rate of 40 kg ha^−1^; there was no increase in biomass beyond this rate. Under partially-weedy conditions, however, rice biomass increased with each increment in the seeding rate past 20 kg ha^−1^. This was probably because at low crop densities, there is less canopy cover early in the growing season, leaving more resources available for the weeds and thus enabling them to establish and grow quickly. At high crop density, the canopy closes quickly and shades out the weeds. In an earlier study on wheat, the highest crop biomass was obtained at a high crop density in the presence of weeds [23]. Under weed-free conditions, the plasticity in crop growth allows them to produce more tillers at low density. Previous studies on wheat [17], barley [16], and rice [26] also reported strong and consistent negative effects of increased crop density on weed biomass, as well as positive effects on crop biomass.

**Table 8:** Effect of rice seeding rate and weed infestation level on rice biomass (g m^−2^) in the aman seasons of 2012 and 2013.

Seed rate (kg ha^−1^)	Rice biomass							
	At 35 days after sowing				At crop anthesis			
	2012		2013		2012		2013	
	Weed-free	Partially-weedy	Weed-free	Partially-weedy	Weed-free	Partially-weedy	Weed free	Partially-weedy
	g m^−2^							
20	22.3	15.2	21.2	11.6	796.9	408.4	937.4	320.1
40	48.5	22.2	34.1	20.3	912.7	472.7	1053.2	435.2
60	52.1	25.4	43.6	28.7	917.4	585.8	1061.5	572.7
80	58.6	33.5	47.4	34.4	897.3	619.9	1037.3	616.3
100	63.6	45.1	50.5	38.4	901.6	655.2	1055.4	632.2
LSD^a^_0.05_	17.4		1.2		123.5		68.9	
LSD^b^_0.05_	4.0		3.7		91.1		64.2	

a LSD  =  compared within the same seeding rate.

b LSD  =  compared within the same level of weed infestation.

Abbreviations: LSD_0.05_, least significant difference at 5% level of significance; NS, non-significant.

### Rice Panicle

**Figure 5:**
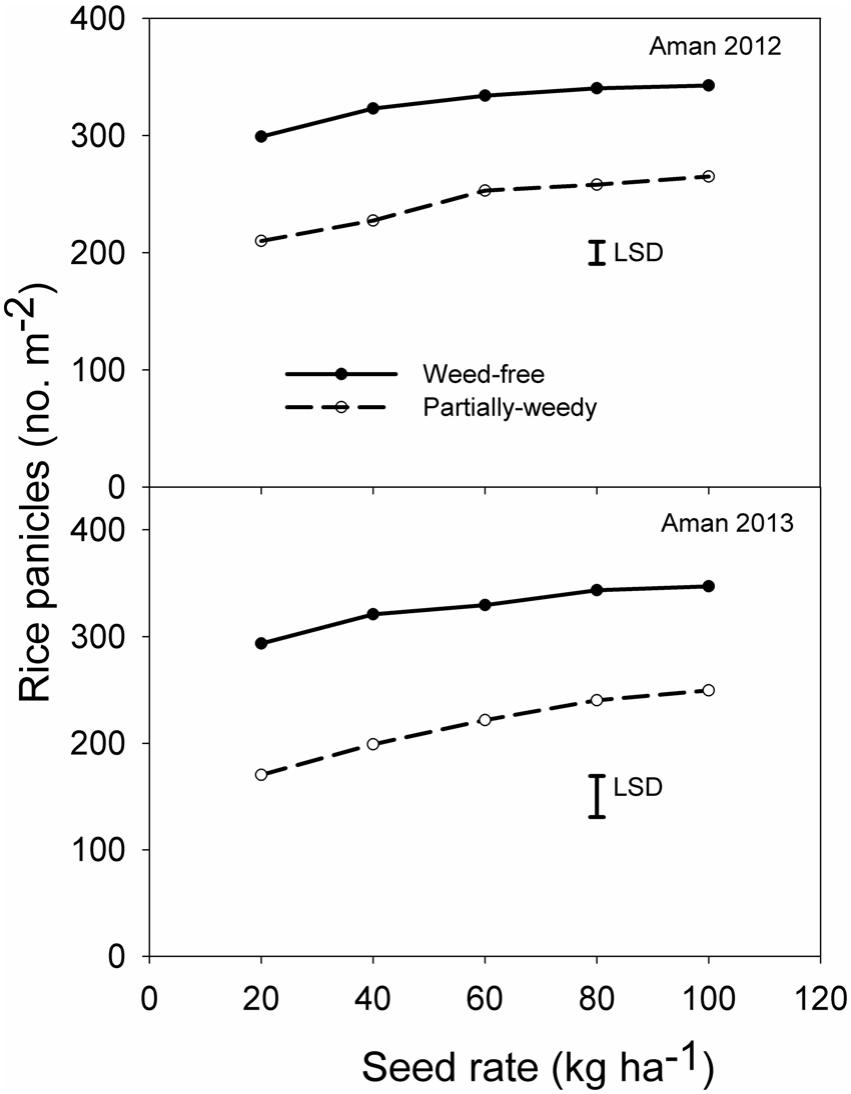
Effect of rice seeding rate and weed infestation level on rice panicle (number m^−2^) at crop harvest in 2012 and 2013.

The interaction between weed infestation level and seeding rate had a significant effect on the number of rice panicles in both seasons (Figure 5). In both partially-weedy and weed-free treatments, panicle numbers increased as seeding rates were increased from 20 kg ha^−1^ to 100 kg ha^−1^. However, increase in panicle number tended to be greater in the partially-weedy plots than in the weed-free plots. Under weed-free conditions, panicle number at 100 kg seed ha^−1^ increased by 15 and 18% in 2012 and 2013, respectively, compared with panicle number at 20 kg seed ha^−1^. Under partially-weedy conditions, these values were 26 and 47%, respectively. In the partially-weedy plots, panicle number decreased by 30 to 36% compared with the weed-free plots. Similar results were observed in an earlier study conducted in the Philippines in which the weed-free treatment posted 50–67% higher panicle numbers than the partially-weedy treatment at 15 kg ha^−1^ seeding rate, while at 100 kg ha^−1^ these values were only 16–25% [27].

### Rice Grains Panicle^−1^

**Figure 6:**
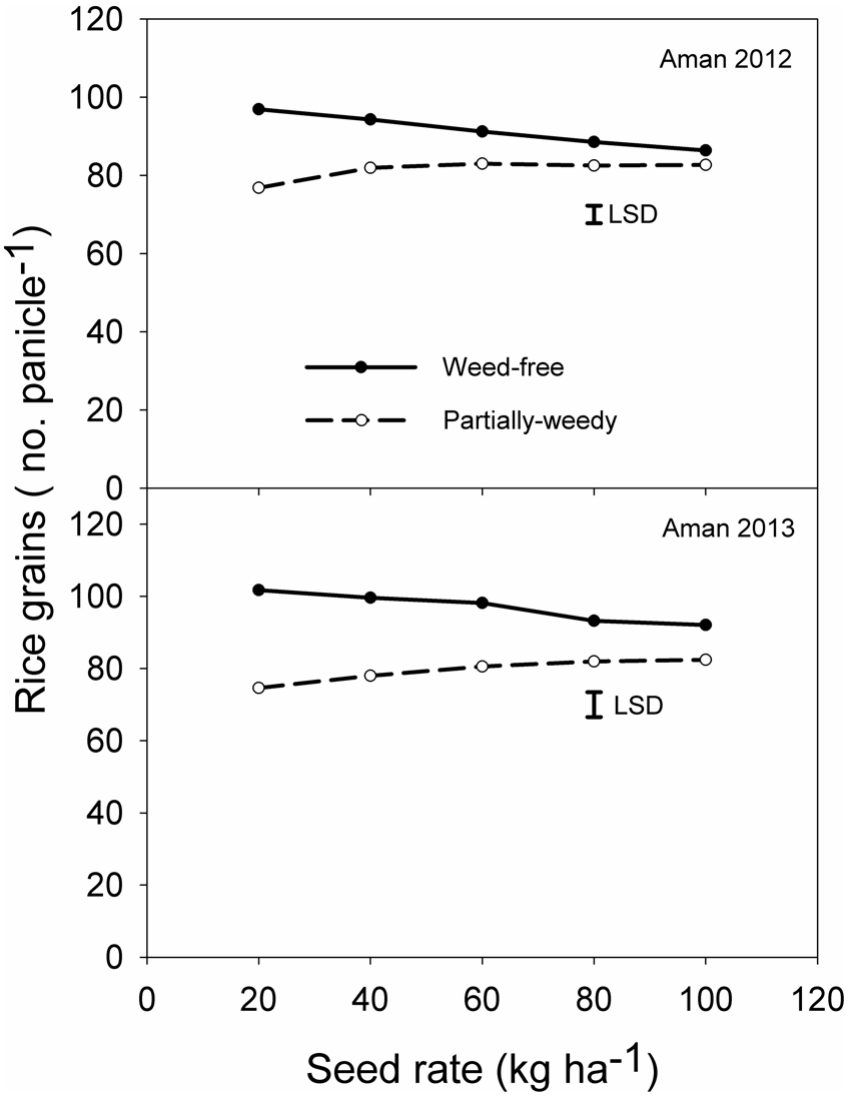
Effect of rice seeding rate and weed infestation level on the number of rice grains panicle^−1^ at crop harvest in 2012 and 2013.

The number of grains panicle^−1^ was influenced by the interaction between weed infestation level and rice seeding rate. Under weed-free conditions, the number of grains panicle^−1^ decreased with increase in seeding rate; however, under partially-weedy conditions, this result was reversed (Figure 6). The greater number of grains panicle^−1^ (97–102) was recorded under weed-free conditions at seeding rate of 20 kg ha^−1^ whereas, under partially-weedy conditions, the greatest number of grains panicle^−1^ (81–83) was recorded at seeding rate of 80 kg ha^−1^. Regardless of the seeding rate, the number of grains panicle^−1^ decreased by 9–11% under weed-free conditions when seed rate increased from 20 to 100 kg ha^−1^. Under partially-weedy conditions, the number of grains panicle^−1^ increased by 7–9%. In a previous study, it was reported that increase in seeding rate from 200 to 400 seeds m^−2^ decreased the number of grains panicle^−1^ by 17% [22].

### Rice Grain Yield

**Figure 7:**
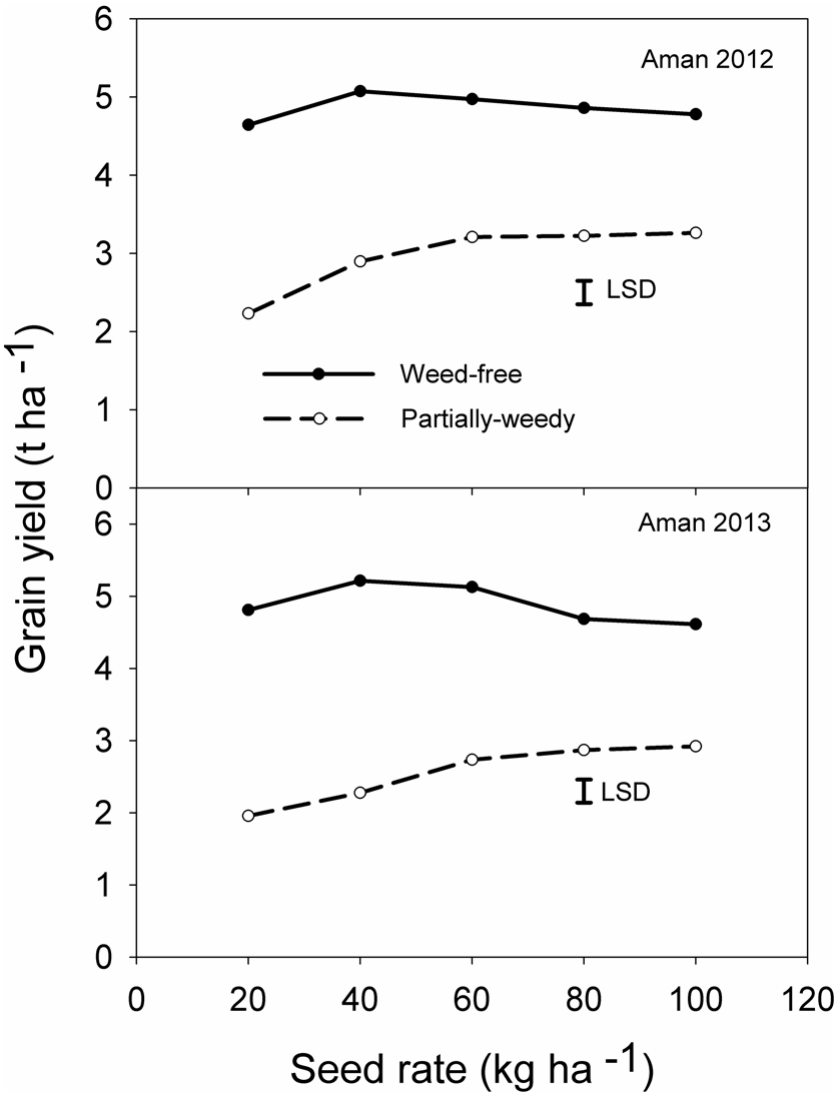
Effect of rice seeding rate and weed infestation level on rice grain yield (t ha^−1^) in 2012 and 2013.

Grain yield was significantly influenced by the interaction between weed infestation level and seed rate (Figure 7). Regardless of the seeding rate, yield was always higher in the weed-free plots (4.6–5.2 t ha^−1^) than in the partially-weedy plots (2.0–3.2 t ha^−1^). In the weed-free plots, the highest yield (5.1–5.2 t ha^−1^) was recorded at a seeding rate of 40 kg ha^−1^ in both seasons. Under these conditions, grain yield increased by 7–8% with increase in seeding rate from 20 to 40 kg ha^−1^; beyond this rate, grain yield declined slightly. Under partially-weedy conditions, however, yield continued to increase across the entire range of seeding rates, whereas in the presence of weeds, yield increased by 30–33% when seeding rate increased from 20 to 100 kg ha^−1^.

Under weedy or partially-weedy conditions, a higher seeding rate suppresses weed growth and increases grain yield. Such results, however, depend on environmental conditions, crop genotypes, and the growing season. In a previous study on wheat, under weed-free environments grain yield ranged from 2.2 to 2.3 t ha^−1^, when seeding rate ranged from 100 to 200 plant m^−2^ and, beyond this, yield declined slightly (by about 0.1 t ha^−1^). However, in the presence of weeds, yield continued to increase (17 and 23% increase when seeding rate increased to 200 and 300 wheat plant m^−2^, respectively, compared with 100 plant m^−2^) across the entire range of densities [21]. In another study, no differences were found in wheat yield at different crop densities in herbicide-treated plots whereas, in weedy plots, grain yield increased by 22% from low to medium and high crop density [28]. Our results also support the findings of another study on aerobic rice in the Philippines, in which the yield of an inbred variety under partially-weedy conditions increased from 0.8 to 2.4 t ha^−1^ (70% yield increase) with increase in seeding rate from 15 to 125 kg ha^−1^ [28].

In DSR systems in Bangladesh, farmers use seeding rates ranging from 50 to 100 kg ha^−1^, depending on season, variety, seed quality, and field conditions. However, there is no specific recommendation available in literature for Bangladesh conditions. Seeding rate is an important factor for optimum yield in DSR. High seeding rate helps to suppress weeds; however, yield does not always increase with high seeding rate and there are some risks associated with this such as crop lodging, diseases, and insect infestation. On the other hand, low seeding rates reduces seed cost. However, there are also some risks associated with the use of low seeding rate such as losses due to weed competition and poor rice seedling establishment [29]. Therefore, seed price, seed quality, field situations, and chances of seed loss by insects, and diseases need to be considered before recommending a seeding rate [28].

The present study showed that seeding rate of 40 to 60 kg ha^−1^ in the weed-free and 80 to 100 kg ha^−1^ in the partially-weedy condition resulted in better crop. Under weed-free conditions, yield increased up to the seeding rate of 40 kg ha^−1^ and decreased slightly beyond this rate. In the partially-weedy plots, grain yield continued to increase up to the highest tested seeding rate (i.e., 100 kg ha^−1^), mainly because increasing crop density suppressed weed growth and reduced losses in grain yield. Our results are also supported by those of an earlier study, in which increasing rice seeding rates suppressed weed growth and reduced losses due to weeds [19].

Weeds are the major constraint to the success of DSR. In our study, the average yield loss under partially-weedy conditions ranged from 40 to 48% compared with that under weed-free conditions, which confirms that weeds are a crucial yield-limiting factor in DSR and that weed management should be properly addressed to make DSR cultivation more profitable.

Due to labor scarcity and its high cost, herbicides are considered the best option for managing weeds in DSR. But sole dependence on herbicides is not sustainable because of its harmful effect on the environment and toxicity risk to crops if not applied at optimum dose. In addition, excess use of herbicides can result in the evolution of resistant biotypes. Weed management in DSR therefore needs to be considered and integrated with all possible agronomic practices. High seeding rates play an important role in increasing the competitiveness of the crop against weeds, which minimizes weed pressure and reduces dependence on herbicide use in DSR. The results of our study indicate that, in Bangladesh, farmers can use a seeding rate of 40 kg ha^−1^ where weed management is not a problem. However, in the absence of effective weed control, farmers should use relatively higher seeding rates (80 to 100 kg ha^−1^) to reduce yield loss due to weed infestation.
